# B7-H3 in Brain Malignancies: Immunology and Immunotherapy

**DOI:** 10.7150/ijbs.85813

**Published:** 2023-07-24

**Authors:** Xiaopeng Guo, Mengqi Chang, Yu Wang, Bing Xing, Wenbin Ma

**Affiliations:** 1 Department of Neurosurgery, Peking Union Medical College Hospital, Chinese Academy of Medical Sciences and Peking Union Medical College, Beijing 100730, China; 2 Medical Research Center, Peking Union Medical College Hospital, Chinese Academy of Medical Sciences and Peking Union Medical College, Beijing 100730, China.

**Keywords:** B7-H3, brain tumor, tumor microenvironment, cancer immunotherapy, chimeric antigen receptor T cell therapy, antibody-drug conjugate therapy

## Abstract

The immune checkpoint B7-H3 (CD276), a member of the B7 family with immunoregulatory properties, has been identified recently as a novel target for immunotherapy for refractory blood cancers and solid malignant tumors. While research on B7-H3 in brain malignancies is limited, there is growing interest in exploring its therapeutic potential in this context. B7-H3 plays a crucial role in regulating the functions of immune cells, cancer-associated fibroblasts, and endothelial cells within the tumor microenvironment, contributing to the creation of a pro-tumorigenic milieu. This microenvironment promotes uncontrolled cancer cell proliferation, enhanced metabolism, increased cancer stemness, and resistance to standard treatments. Blocking B7-H3 and terminating its immunosuppressive function is expected to improve anti-tumor immune responses and, in turn, ameliorate the progression of tumors. Results from preclinical or observative studies and early-phase trials targeting B7-H3 have revealed promising anti-tumor efficacy and acceptable toxicity in glioblastoma (GBM), diffuse intrinsic pontine glioma (DIPG), medulloblastoma, neuroblastoma, craniopharyngioma, atypical teratoid/rhabdoid tumor, and brain metastases. Ongoing clinical trials are now investigating the use of CAR-T cell therapy and antibody-drug conjugate therapy, either alone or in combination with standard treatments or other therapeutic approaches, targeting B7-H3 in refractory or recurrent GBMs, DIPGs, neuroblastomas, medulloblastomas, ependymomas, and metastatic brain tumors. These trials hold promise for providing effective treatment options for these challenging intracranial malignancies in both adult and pediatric populations.

## Introduction

Immune checkpoints (ICs) are a group of antigens that deliver signals that either enhance or suppress immune responses to regulate the activation, direction, and magnitude of host immune responses. Well-known inhibitory ICs, such as programmed cell death protein 1 (PD-1), programmed cell death ligand 1 (PD-L1), and cytotoxic T lymphocyte-associated protein 4 (CTLA-4), can induce T cell exhaustion and death, contributing to the suppression of anti-tumor immunity and subsequent cancer progression, making them novel therapeutic targets for anti-tumor immunotherapy 1. B7 family are a group of widely studied immunomodulatory ligands that activate or inhibit the function and proliferation of effector immune cells 2. These molecules are usually exerting an immune-suppressing effect and are expressed on the surface of both immune cells and cancer cells. They contribute directly to tumor progression or indirectly by influencing the anti-tumor immune system. A total of 10 members of the B7 family have been identified, including B7-1 (CD80), B7-2 (CD86), B7-DC (CD273/PD-L2), B7-H1 (CD274 or PD-L1), B7-H2 (CD275), B7-H3 (CD276), B7-H4 (B7x/B7S1/VTCN1), B7-H5 (GI24/VISTA/PD-1H), B7-H6 (NCR3LG1), and B7-H7 (HHLA2) 2. Since its discovery in 2001, B7-H3 has garnered significant attention 3. However, its biological function in the immune system is complex and still being investigated. While some studies have reported its ability to enhance immune function, most studies have found that B7-H3 contributes to tumorigenesis, angiogenesis, invasion, and metastasis through complex mechanisms of action 4. Blocking or silencing B7-H3 has been shown to eliminate tumors and prolong survival in hematological malignancies and some solid tumors 5.

Patients with brain metastasis, glioblastoma (GBM), or diffuse intrinsic pontine glioma (DIPG) has the most dismal prognosis. Brain metastases make up the majority of intracranial neoplasms and develop when cancer cells from other organs spread through the bloodstream and establish themselves in the brain. Among primary malignant brain tumors in adults, GBM is the most common 6, while DIPG, a high-grade glioma originating from the brainstem, accounts for 10% of all pediatric tumors 7. Despite remarkable progress made in conventional treatments such as surgery, radiotherapy, and chemotherapy for these brain malignancies, prognosis remains poor compared with other cancers 8. Although postoperative radiochemotherapy and adjuvant chemotherapy with temozolomide, tumor treating fields, and DCVax-L have shown significant survival benefits in GBMs, research on targeted immunotherapies has unfortunately yielded limited benefits on patient survival over the past two decades 9-11. However, with the rapid advancement and valuable experience of research on B7-H3 immunology and successful application of immunotherapy targeting B7-H3 in blood cancers and certain advanced solid cancers such as gastrointestinal cancers, ovarian cancers, renal cancers, and liver cancers 12-18, studies investigating the potential of B7-H3 as a treatment for brain tumors, though limited, deserve greater attention and recognition.

A comprehensive and up-to-date review is needed to enhance our understanding on the immunology and immunotherapy of B7-H3 in brain malignancies. Thus, in this review, we made a detailed summary by comprehensively reviewing the latest research progress on the structure and biological functions of B7-H3, its interactions with the tumor microenvironment (TME) components, its pro-tumorigenesis roles and underlying mechanisms of action, current research on B7-H3-related immunology and immunotherapy in different brain tumor types, and promising ongoing clinical trials exploring the anti-tumor efficacy and safety of B7-H3 targeted immunotherapies for brain malignancies.

## Structure and Ligands of B7-H3

### Structure, expression, and existing forms of B7-H3

**Figure 1:**
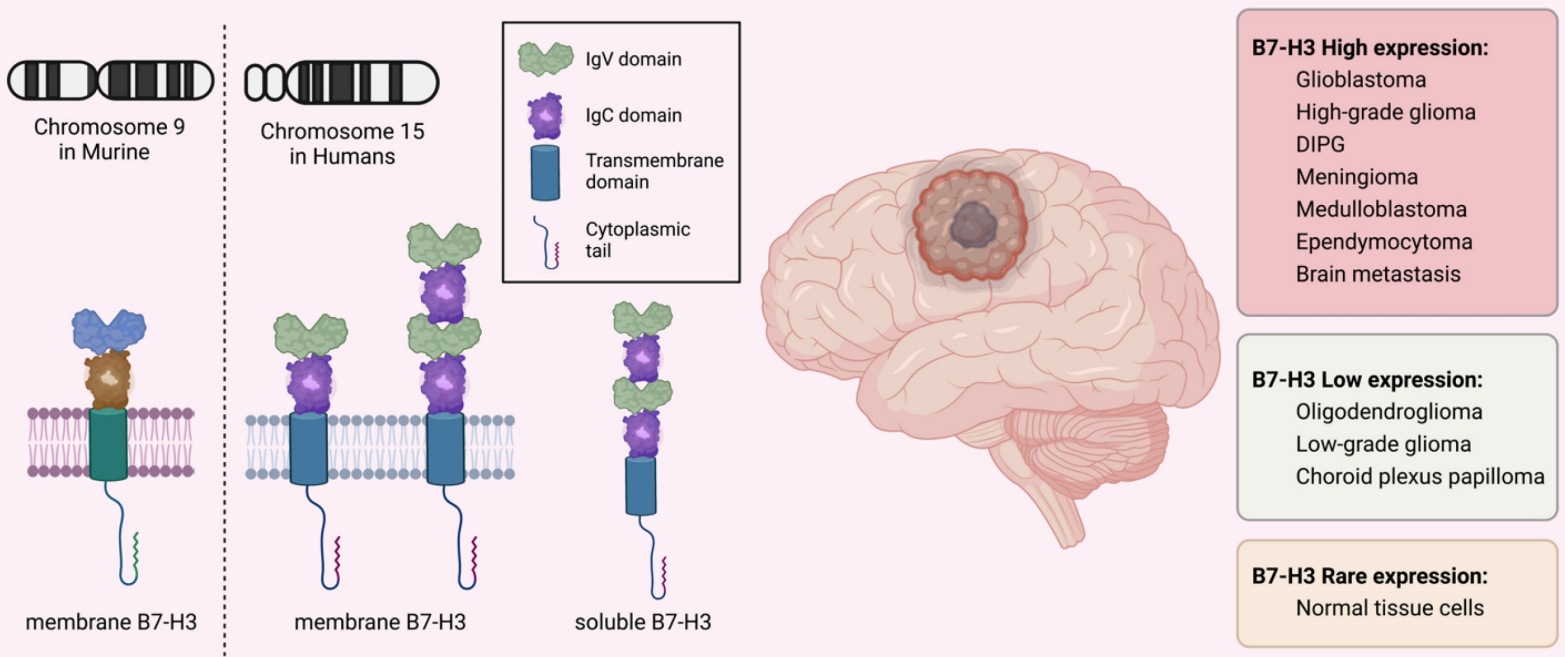
** Structure and Expression of B7-H3 (CD276).** B7-H3 is encoded by the exons located on chromosome 9 in mice and on chromosome 15 in humans. The molecule consists of a short cytoplasmic tail, a single transmembrane sequence, and extracellular immunoglobulin domains. In mice, the extracellular domain is composed of an N-terminal IgV and a C-terminal IgC, while in humans, the predominant isoform is 4IgB7-H3, which contains two duplicated IgV-IgC domains. B7-H3 expression varies among different brain tumors but is rarely expressed on normal cells. In addition to the membrane-bound form, the soluble form of B7-H3 has also been detected in human plasma.

The immune checkpoint B7-H3 is a type I transmembrane glycoprotein consisting of 316 amino acids encoded by 12 exons located in chromosome 9 in murine while in 15q24.1 in humans 19. It consists of a cytoplasmic tail of 45 amino acids, a single transmembrane segment, and extracellular immunoglobulin domains. In mice, the extracellular domain is made up of an N-terminal IgV and a C-terminal IgC domain, while in humans, there are two isoforms with differing pairs of Ig ectodomains: 2IgB7-H3 (B7-H3 VC) with 93% amino acid similarity to murine 2IgB7-H3 and 4IgB7-H3 (B7-H3 VCVC or B7-H3b), which contains two tandemly duplicated IgV-IgC domains 20-22. The 4IgB7-H3 isoform is the predominant one in humans and is extensively expressed on the surface of cancerous cells and immune cells (**Figure 1**).

B7-H3 is expressed in various types of brain tumors including GBM 23, 24, high-grade gliomas 25-27, DIPG 28, pleomorphic xanthoastrocytoma 29, meningioma 30, 31, craniopharyngioma 32, 33, atypical teratoid/rhabdoid tumors (ATRT) 34, medulloblastoma 35, 36, and neuroblastoma 37. However, the level of its expression varies among different brain tumor types. The expression of B7-H3 is moderate-to-high in GBM, high-grade glioma, DIPG, meningioma, medulloblastoma, and ependymocytoma, while is lower in choroid plexus papilloma, oligodendroglioma, and low-grade glioma 26-28, 35, 38, 39. The expression of different B7-H3 isoforms varies at different disease stages in GBMs. Specifically, 4IgB7-H3 is detected only in newly-diagnosed GBM cells, while 2IgB7-H3 is predominantly detected in recurrent GBM cells. This provides clinical implications for targeting 4IgB7-H3 in newly-diagnosed GBMs while using 2IgB7-H3 to track recurrence and treatment responses 23. B7-H3 mRNA is present in most normal cells, but its expression levels are typically low. This is in stark contrast to its high expression in cancer cells 40-42.

The crystal structure of B7-H3 has been reported and the FG loop of IgV domains is the structural basis for its function 41. Aside from the most extensively studied type of B7-H3 found on the cell membrane (mB7-H3), a "soluble" form of B7-H3 (sB7-H3) has also been detected in plasma 43. This form of B7-H3 is produced through an alternative splicing process from the fourth intron of the B7-H3 gene, which is mediated by matrix metalloproteinase, and is commonly released from monocytes, monocyte-derived dendritic cells (DCs), activated T lymphocytes, and mB7-H3-positive cancerous cells 43, 44. Nevertheless, the existence of sB7-H3 remains uncertain due to the lack of evidence regarding its solubility and expression in exosomes 19, 45.

### Ligands of B7-H3

**Figure 2:**
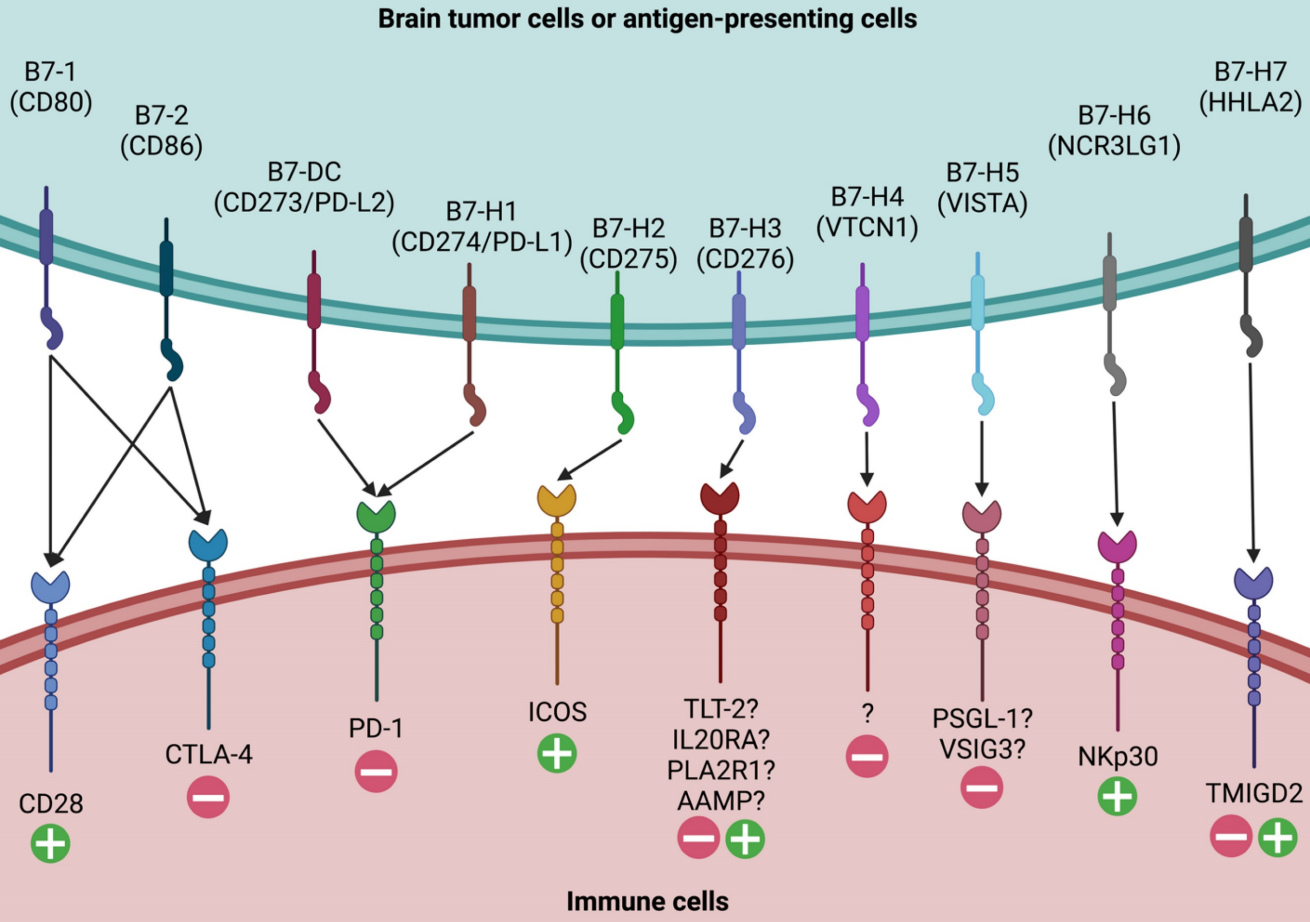
** Ligands for B7 Family Members.** Ten members of the B7 family have been identified, including B7-1, B7-2, B7-DC, B7-H1, B7-H2, B7-H3, B7-H4, B7-H5, B7-H6, and B7-H7. While major receptors for the majority of B7 family molecules have been identified, the putative ligands for B7-H3 have not been clearly elucidated. Four candidates, including TLT-2, IL20RA, PLA2R1, and AAMP, have been proposed as the possible binding receptors for B7-H3 and need further validation.

Given the complex and context-specific functions of B7-H3 as both a co-stimulatory and co-inhibitory molecule, it is crucial to identify its binding receptors and understand their interactions. Unlike its superfamily members such as B7-1, B7-2, PD-L1, PD-L2, and B7-H2, which have known ligands like CD28, CTLA-4, PD-1, and inducible T cell costimulatory, the ligands for B7-H3 have yet to be clearly identified (**Figure 2**).

Four candidates have been proposed as possible binding receptors for B7-H3: triggering receptor expressed on myeloid cells (TREM)-like transcript 2 (TLT-2), interleukin-20 receptor subunit α (IL20RA), phospholipase A2 receptor 1 (PLA2R1), and angio-associated migratory cell protein (AAMP) 46-49. TLT-2, a single transmembrane protein, was the first identified B7-H3 ligand candidate expressed in macrophages, microglia, neutrophils, and T/B lymphocytes 46, 50, 51. It contains a putative SH3 binding motif and has multifaceted functions in modulating both the innate and adaptive immune responses 52-54. However, the binding of B7-H3 and TLT-2 has been disputed since subsequent studies have failed to provide solid evidence to support their interactions in both murine and human cells 55, 56. IL20RA, a transmembrane glycoprotein with two tandem β-sandwich extracellular domains, has been recently reported as another putative binding target of B7-H3 using high-throughput interactome platforms 47, 48. It is expressed in the skin, lung, and testis, with low expression levels in circulating immune cells 57, 58. Studies have reported that the B7-H3-IL20RA binding may have indirect functions in modulating immune responses, potentially via stromal substances or cancer cells 57-59. PLA2R1, a transmembrane protein with a short cytoplasmic tail, a transmembrane sequence, a tandem C-type lectin domain, a fibronectin type II domain, and a cysteine-rich terminal domain, is another high-affinity B7-H3 binding receptor 48. However, the mechanism by which the B7-H3-PLA2R1 partner regulates immune responses remains to be elucidated. Recently, AAMP was discovered as another novel binding partner 49. Researchers have demonstrated that AAMP expressed on glioma cells and immune cells is the binding receptor and has a synergistic effect with B7-H3. The immunosuppressive function of B7-H3 can be down-regulated by targeting AAMP.

## Immunological and Non-immunological Functions of B7-H3

### Functions through the innate and adaptive immunity

The functions of B7-H3 in modulating innate immunity are multifaceted and intricate. It has been found to enhance both lipopolysaccharide- and bacterial lipoprotein-induced NF-kappaB activation, and promote the release of pro-inflammatory cytokines in a toll-like receptor (TLR)-2- and TLR-4-dependent manner. However, it fails to stimulate inflammatory responses in murine macrophages and inhibits natural-killer (NK) cell activation 60. In humans, high levels of sB7-H3 in the plasma are correlated with the levels of plasma TNF-alpha and IL-6, and predict poor outcomes in patients with severe sepsis 60.

In adaptive immunity, B7-H3 acts as both a co-stimulator and a co-inhibitor of T cell-mediated immune responses. Initial research has shown that B7-H3 has co-stimulatory effects on the immune system 3. It increases the proliferation of both CD4+ and CD8+ T cells, stimulates the production of IFN-γ, and enhances cytotoxic T cell activity. Following studies have revealed that B7-H3 leads to the eradication of tumors and the reduction of metastasis through the rapid expansion of tumor-specific CD8+ cytotoxic T cells and NK cells, and increased production of IL-12 61-63. B7-H3 also mediates the development of acute and chronic allograft rejection by stimulating T-cell immune responses and promoting the production of cytokines and chemokines 64. In addition, B7-H3 promotes collagen-induced arthritis and autoimmune encephalomyelitis in murine models by stimulating the activity of T helper type 1 (Th1)/Th17 cells and activating the production of IFN-γ and IL-17 65. However, these results have rarely been validated in humans. The lack of B7-H3 binding to TLT-2 and the expression of 4IgB7-H3 isoforms in humans may explain this difference between mice and men.

Contrary to its co-stimulatory immune functions, more recent studies suggest that B7-H3 inhibits T-cell activation and proliferation, thereby negatively regulating adaptive immune responses in both cancerous and non-cancerous conditions. Studies have demonstrated that B7-H3 inhibits cytokine production through signaling pathways involving NFTA, NF-kappaB, and AP-1 factors, which regulate gene transcription and immune cell proliferation 66-68. *In vivo* studies have further validated the inhibitory function of B7-H3. B7-H3-knockout mice develop severe asthma with high concentrations of IL-5 and IL-13, eosinophil infiltrations in the lungs, and elevated anti-ovalbumin antibodies in the plasma, indicating the co-inhibitory function of B7-H3 on Th2-cell responses 65. In allograft models, blocking B7-H3 using monoclonal antibodies results in enhanced IFN-γ production and accelerated rejection, further suggesting the co-inhibitory role of B7-H3 69. In cancerous models, B7-H3 has been reported to promote tumorigenesis by inhibiting T-cell mediated anti-tumor immune functions. The expression level of B7-H3 in tumors correlates with a low concentration of infiltrating T lymphocytes, elevated regulatory T (Treg) cell infiltration, and suppression of NK-cell-mediated tumor cell lysis process, leading to accelerated cancer cell proliferation and mobility 70-72.

Although numerous investigations have provided evidence for the multifaceted biological functions of B7-H3 in both innate and adaptive immunity in cancerous and non-cancerous settings as discussed above, discrepancies and controversies still exist 2, 73, 74. One point is that B7-H3 interact with both stimulatory and inhibitory ligands with varied binding affinity, and its final effects are determined by the comprehensive summation of multiple downstream signaling pathways activated by different ligand-receptor partners. Another factor is that the expression level of B7-H3 varies substantially among different tissues and disease models, which may reshape the immune-modulatory functions of B7-H3 and result in tissue- and disease-specific outcomes. Intratumoral heterogeneity, complex tumor microenvironmental context composites, and imbalanced signaling intensity are also potential factors responsible for the dual functions of B7-H3 4, 19, 73.

### Functions through non-immunological mechanisms

**Figure 3:**
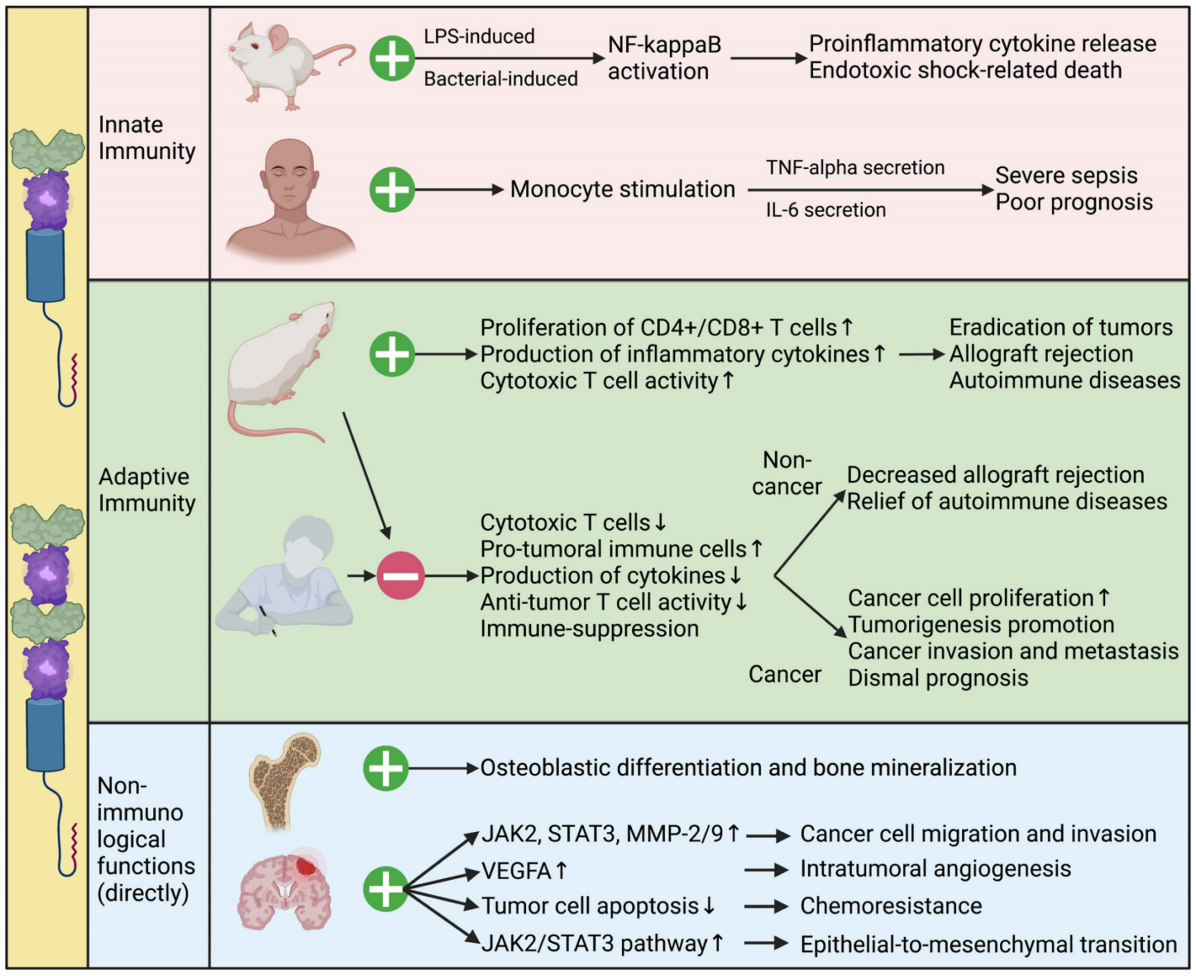
** Immunological and Non-immunological Functions of B7-H3.** Functions of B7-H3 in modulating tumorigenesis, sepsis, allograft rejection, autoimmune diseases, and osteogenesis via immunological and non-immunological mechanisms are complex and multifaceted, as investigated in murine models and patients. Further exploration is under way.

Apart from its immunological functions, B7-H3 also plays important non-immunological roles in both normal and cancerous tissues (**Figure 3**). During embryogenesis, B7-H3 is highly expressed on developing bones and plays a critical role in osteoblastic differentiation and bone mineralization. Inhibition of B7-H3 using B7-H3-Fc suppresses this process 75. In cancer, B7-H3 has been implicated in metastasis, angiogenesis, and tumorigenesis through various non-immunological mechanisms 12, 76, 77. Studies have shown that B7-H3 promotes cancer cell migration and invasion by regulating metastasis-associated proteins such as Janus kinase 2 (JAK2), signal transducer and activator of transcription 3 (STAT3), matrix metalloproteinase (MMP)-2, and MMP-9. It also promotes intratumoral angiogenesis by upregulating the expression of vascular endothelial growth factor A (VEGFA) 12, 78, 79. Moreover, B7-H3 down-regulates tumor cell apoptosis, reduces G2/M-phase arrest, and thus decreases the sensitivity to anti-tumor drugs, leading to increased chemoresistance 80-83. Recent studies have also demonstrated that B7-H3 promotes epithelial-to-mesenchymal transition (EMT) via the JAK2/STAT3 pathway and stimulates cancer cell glycolytic metabolism 17, 84, 85.

## Interactions of B7-H3 with the TME components

**Figure 4:**
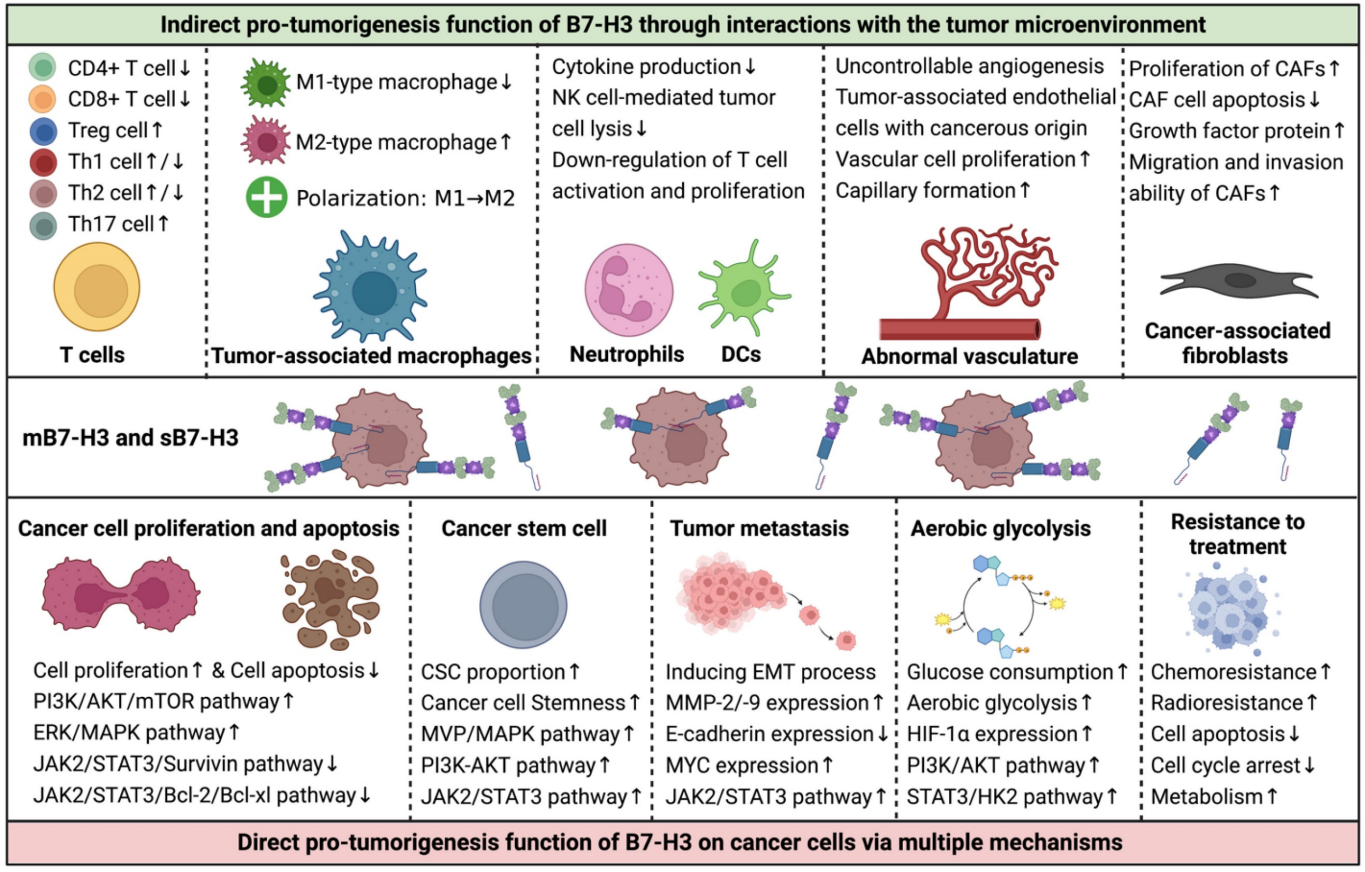
** Interactions of B7-H3 with the Tumor Microenvironment (TME) and Its Effects on Tumor Cells.** Although the functions of B7-H3 in regulating tumor behaviors are complex, the majority of studies suggest that it plays pro-tumorigenic roles in assisting the development and progression of cancers. B7-H3 exerts its effects mainly in two ways: 1) indirectly, via communications with components of the TME, including infiltrated immune cells, aberrant vascular networks, and cancer-associated fibroblasts, and 2) directly, via multiple signaling pathways that regulate cancer cell proliferation, apoptosis, stemness, invasion, migration, metabolism, and treatment responses.

The TME is a complex and dynamic structure that includes infiltrating immune cells, stromal components, vascular networks, extracellular matrix, exosomes, cytokines, and chemokines, in addition to malignant cells 86. The interactions between tumor cells and the TME play a critical role in tumor initiation, progression, metastasis, and therapeutic responses. B7-H3 plays a significant role in tumor biology by modulating the functions of infiltrating immune cells, angiogenesis, and cancer-associated fibroblasts (CAFs) in the TME (**Figure 4**) 17, 70, 87-93.

### B7-H3 and infiltrating immune cells

The major infiltrating immune cells include T cells, TAMs, DCs, NK cells, and neutrophils, which exert both pro- and anti-tumor functions. For instance, CD4+ Th cells, CD8+ cytotoxic T cells, and M1-type TAMs promote cancer-killing functions, while Treg cells and M2-type TAMs suppress anti-tumor immunity and enhance tumorigenesis 86, 89, 94-96.

T lymphocytes are a significant group of adaptive anti-tumor immune cells that infiltrate the TME. Different T cell types perform specific immune functions. Studies have shown that B7-H3 inhibits the proliferation and activity of CD4+ and CD8+ effector T cells, leading to immunity inhibition and tumor progression. Blocking B7-H3 reverses the state of immunosuppression, restores anti-tumor immunity, and creates an anti-tumorigenesis TME 2, 19, 20, 41, 55, 66, 70, 72, 73. Treg cells represent approximately 7% of CD4+ T lymphocytes and express CD25 and forkhead box P3 (FOXP3), which promotes immune tolerance and helps in cancer immune evasion 94. CD4+CD25+ Treg cells up-regulate B7-H3 expression and down-regulate MHC-peptide complexes on antigen-presenting DCs, leading to suppressive function in stimulating T-cell-mediated immune responses 95. Another study indicated a positive correlation between FOXP3+ Treg cells and B7-H3 expression on tumor cells, suggesting a possible cooperative relationship between B7-H3 and Treg cells in cancer immune evasion 71. Th cells, which differentiate from naïve CD4+ T cells, produce lineage-specific cytokines and play crucial roles in coordinating immune responses to infections and autoimmune diseases 96. Th cell differentiation is determined by T-cell receptor signaling and other triggering signals, such as those from B7-H3. In a murine model of autoimmune encephalomyelitis, B7-H3-deficient mice experienced more severe inflammation, and Th cells differentiated preferentially toward Th1 rather than Th2 cells, indicating the stimulatory function of B7-H3 for Th2 cells 67. However, in another model of allergic conjunctivitis, B7-H3 negatively regulated both Th2 and Th1 immune responses 97. Further conflicting results have shown that B7-H3 in murine models with autoimmune diseases has co-stimulatory functions for Th1 and Th17 cells but co-inhibitory functions for Th2 cells 65. The contradictory immune effects of B7-H3 on Th cells may arise from the analysis of varied disease settings, highlighting the need for further investigation and validation.

TAMs are crucial immune cells infiltrating the TME and exist in two polarization states: M1-type macrophages that enhance inflammation and promote anti-tumor immune functions, and M2-type macrophages that play a critical role in immune down-regulation and tumor evasion 89. The polarization of TAMs is constantly in flux and determined by the prevailing signals in the TME. TAMs express high levels of B7-H3, which exhibits pro-metastatic and immunosuppressive functions by promoting tumor angiogenesis and extracellular matrix reconstruction, dampening T-cell infiltration, and suppressing immune responses 92. Additionally, B7-H3 on cancerous cells and antigen-presenting cells promotes the polarization of TAMs from M1- to M2-macrophages, impairing anti-tumor immune functions and mediating tumor progression 98-101.

Neutrophils and DCs are also prominent components of the TME that express high levels of B7-H3. B7-H3 on DCs and neutrophils correlates with reduced IL-12 production and down-regulated T cell activation and proliferation, resulting in an immune-inhibitory effect and promoting a pro-tumorigenesis TME 74, 102. Both the cell-binding m4IgB7H3 and the supernatant s4IgB7H3 of glioma samples are functional and significantly suppress NK cell-mediated tumor cell lysis; Silencing B7-H3 in cells makes them more susceptible to NK cell-induced cytotoxicity than unsilenced cells 72.

B7-H3 plays a pivotal role in communicating with multiple immune infiltrates, regulating the activation of immune cells and production of cytokines and chemokines, dictating the features of the TME, and influencing the biology of cancers. In addition, B7-H3 affects immune cells differently and has varied influences on the same immune cells in different cancers, contributing to the complex relationships between B7-H3 and infiltrating immune cells in the TME.

### B7-H3 with tumor vasculature and CAFs

Vascular networks in tumors are abnormal and uncontrollable, and angiogenesis is a hallmark of cancer that facilitates improved delivery of oxygen and nutrients, as well as providing a path for tumor metastasis 103. Research has shown that over half of the endothelial cells in GBMs carry the same genomic alterations as GBM cells, indicating that a large portion of vascular networks in the TME have a cancerous origin 93. Moreover, CD133+ stem-like cells can differentiate into endothelial progenitor cells. Inhibition of γ-secretase or silencing NOTCH1 blocks the transition of CD133+ cells into endothelial progenitor cells, while blocking VEGF inhibits the subsequent maturation of endothelial progenitors into tumorous endothelium 104. B7-H3 is overexpressed in tumor-associated endothelial cells and is related to cancer angiogenesis, whereas it is not expressed in normal angiogenic tissues 105. High-grade gliomas are characterized by more aggressive biological behaviors and have higher expression levels of B7-H3 on both the tumor cells and endothelial cells than low-grade gliomas 27, 72, 84. Through the NF-kappaB signaling pathway, B7-H3 accelerates vascular cell proliferation and capillary formation by enhancing the production of VEGF 12. Furthermore, B7-H3 can promote cancer progression and metastasis by accelerating the formation of tumor-associated vessels mediated by CD14+ monocytes 18. MMP-9 and MMP-2 are also involved in B7-H3-mediated cancerous angiogenesis through the PI3K/AKT/MMP pathway 14, 106, 107.

CAFs that comprise a predominant part of the TME also prompt tumor progression and immune escape, in addition to infiltrating immune cells and aberrant vascular networks 91. B7-H3 has a co-stimulatory effect on CAFs, and knockdown of B7-H3 inhibits the proliferation of CAFs, increases CAF cell apoptosis, decreases the expression of stromal cell-derived factor-1 and hepatocyte growth factor protein, and thus promotes tumor progression 90. Moreover, a study has found that B7-H3 is expressed on both the tumor cells and CAFs, and this expression correlates with the migration and invasion ability of CAFs, the secretion of IL-6 and VEGF, and a poor overall prognosis 108.

## Mechanisms of B7-H3 in Promoting Tumorigenesis

### Cancer cell proliferation and apoptosis

B7-H3 is known to promote cancer cell proliferation and inhibit cancer cell apoptosis, leading to sustained tumor growth and progression 70, 81, 83, 84, 107. In addition to impairing anti-tumor immune functions, B7-H3 promotes cancer cell proliferation through the activation of several key signaling pathways via the PI3K or MVP/MEK axes 109, 110. B7-H3 also exerts its anti-apoptotic effects through the JAK2/STAT3 and CDC25A signaling pathways 16, 81, 82, 84, 107. Confirmatory studies have shown that silencing B7-H3 reduces cancer cell proliferation and enhances cancer cell apoptosis by decreasing the expression of several anti-apoptotic and cell cycle-related proteins 16, 80, 111, 112.

### Cancer stem cells

Cancer stem cells (CSCs) or tumor-initiating cells with pluripotent and self-renewing properties are considered the key drivers of tumorigenesis and major sources of treatment resistance and tumor relapse 87. The regulation of CSCs in gliomas is influenced by genetic alterations, epigenetic factors, TME interactions, niche factors, metabolism, and the immune system 87. B7-H3 is significantly overexpressed in CSCs compared to non-stem cancer cells 87, 109, 110, suggesting that targeting B7-H3 may be a promising therapeutic strategy for cancers enriched with B7-H3+ CSCs. Studies have revealed that B7-H3 overexpression increases the proportion of CSC populations through MVP/MAPK, PI3K-AKT, and JAK2/STAT3 signaling pathways and also enhances cancer cell stemness, whereas down-regulation of B7-H3 inhibits CSC functions and stemness 84, 110, 113.

### Tumor metastasis

Metastasis is another essential hallmark of cancer. B7-H3 has been shown to promote glioma cell invasion and induce the EMT process by upregulating MMP-2/-9 expression and downregulating E-cadherin expression 84. In glioma cell and cancer stem cell models, both membrane-bound and soluble forms of B7-H3 promote tumor invasiveness into surrounding brain tissues 72. The JAK2/STAT3/Slug signaling pathway is involved in the EMT process and subsequent tumor invasion and metastasis in gliomas and liver cancers 17, 84, while the PI3K/AKT and MAPK signaling pathways are responsible for EMT in lung cancer and renal cell carcinoma 114, 115. B7-H3 also upregulates MYC expression, leading to GBM cell differentiation and induction of tumor migration and invasion 116.

### Tumor metabolism

Abnormal cancer metabolism, characterized by a shift toward aerobic glycolysis instead of oxidative phosphorylation, fuels tumor evasion, metastasis, and chemotherapy resistance 88. Studies have revealed that B7-H3 promotes glucose consumption, accelerates aerobic glycolysis, and increases lactate production via stabilizing and upregulating hypoxia inducible factor-1 alpha (HIF-1α) expression and its downstream targets that are mediators of the glycolytic pathway through the PI3K/AKT/mTOR pathway and via activating STAT3 and the subsequent HK2, providing new perspectives of immunomodulation function of B7-H3 on tumor progression 117-119. In GBMs, expression of B7-H3 is upregulated in areas with hypoxia 120.

## Immunotherapeutic Strategies Targeting B7-H3

**Figure 5:**
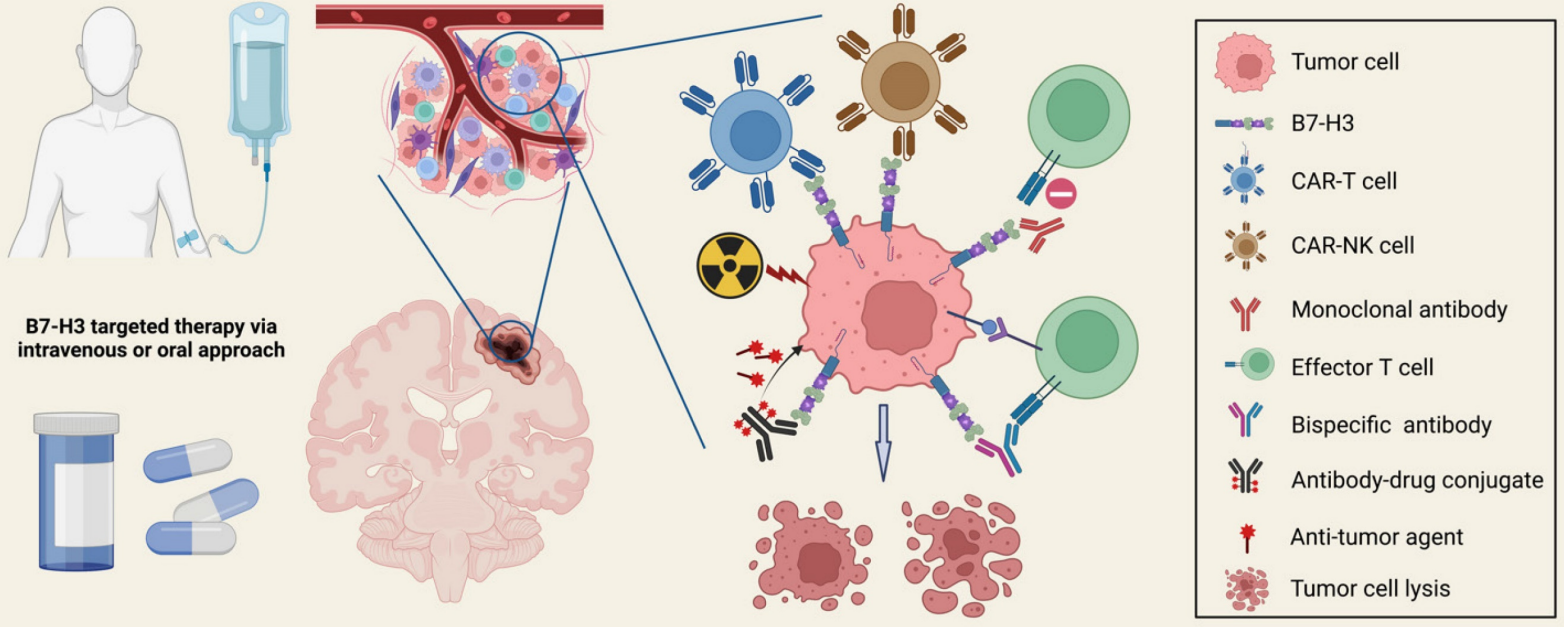
** Therapeutic Strategies Targeting B7-H3.** Multiple preclinical studies and early-phase clinical trials have been investigating novel strategies targeting B7-H3, including CAR-T therapy, CAR-NK therapy, monoclonal antibody blocking, bispecific antibody therapy, antibody-drug conjugate (ADC) therapy, and combination treatments with anti-B7-H3 therapies. These strategies have demonstrated remarkable anti-tumor efficacy, improved clinical outcomes, and acceptable drug-related toxicity.

B7-H3 targeted therapies have been increasingly conducted for malignant brain tumors during the past decade. Recent advances in molecular biology and engineering have paved the way for various therapeutic strategies targeting B7-H3, including chimeric antigen receptor T cell (CAR-T) therapy, CAR-NK cell therapy, antibodies blocking, and antibody-drug conjugates therapy (**Figure 5**). Combining anti-B7-H3 therapies with other treatments is also a promising option for refractory cancers. Most of the aforementioned strategies have undergone verification in preclinical *in vitro* and *in vivo* studies, generating promising safety and anti-tumor efficacy data, and anti-B7-H3 clinical trials are now in progress.

### CAR-based therapy

A CAR is a protein consisting of an extracellular antigen-binding domain, a transmembrane domain that anchors the CAR structure to the effector cell membrane, and an intracellular signaling-activation domain. CAR-T therapy employs autologous or allogeneic T lymphocytes that have been genetically modified with CARs targeting a specific tumor antigen that plays a crucial role in tumor progression, enabling them to effectively recognize and eliminate targeted cancer cells independently of the major histocompatibility complex (MHC) antigen 121.

CAR-T therapy has shown remarkable success in the treatment of hematological malignancies such as refractory acute lymphoblastic leukemia. B7-H3 targeted CAR-T therapy has demonstrated acceptable tolerance and promising effectiveness in GBMs and several other brain cancers 24, 25, 34, 42, 122-124, with many phase-I/II trials still underway. Since B7-H3 is highly expressed on tumor cells, aberrant vessels, and CAFs while rarely on normal cells, B7-H3-CAR-T therapy can directly target cancer cells and disrupt stroma cells and angiogenesis simultaneously without damaging surrounding normal brain tissues.

Studies have shown that B7-H3-CAR-T therapy exerts potent anti-tumor efficacy in pediatric medulloblastoma 122, pediatric glioma 25, cerebral ATRTs 34, DIPG 124, non-small cell lung cancer (NSCLC) brain metastases 125, craniopharyngioma 32, and GBMs 24, 126, 127. In a medulloblastoma xenograft model, B7-H3-CAR-T cells effectively crossed the blood-brain barrier (BBB), mediated anti-tumor immune effects, and induced tumor regression 122. In a study using orthotopic xenografts of pediatric glioma of different grades, both local and systemic B7-H3-CAR-T therapy induced tumor regression and a significant survival advantage 25. Incurable pediatric ATRTs were treated with intracerebroventricular or intratumoral B7-H3-CAR-T cell administration in mouse models, which exhibited potent anti-tumor effects and reduced systemic inflammatory cytokine production 34. The first-in-human phase I trial administering locoregional B7-H3-CAR-T cells to pediatric recurrent/refractory CNS tumors and DIPGs was published in 2023. Three patients with DIPGs were evaluated and preliminary tolerability was witnessed. Intracranial immune activation was correlated with the persistent existence of B7-H3-CAR-T cells in the cerebrospinal fluid 124. Metastatic NSCLC to the brain has an extremely poor prognosis. B7-H3-CAR-T therapy exhibits anti-tumor activity *in vitro* against NSCLC cells and organoids, and in xenotransplant models against orthotopic and metastatic NSCLC 125. Researchers revealed that co-expression of CCR2b in B7-H3-CAR-T cells significantly improved the capability of passing the BBB and enhanced anti-tumor immune effects against brain metastases. In craniopharyngioma with high expression of B7-H3, studies have shown that B7-H3-CAR-T cells exerts tumor-killing effects in the 2D model 32. In GBMs, B7-H3-CAR-T therapy enhances anti-tumor activities, releases effector cytokines, and effectively controls the growth of tumor cell lines and neurospheres 24, 126. Although CD28-co-stimulated B7-H3-CAR-T cells released more inflammatory cytokines, no additional anti-tumor efficacy was observed. Although B7-H3-CAR-T therapy effectively attenuates tumor growth in various xenografts cancer models, it is not able to completely eradicate tumors 5. Spatio-temporal tumor heterogeneity, limited transmission through the BBB, and the immunosuppressive TME are challenges that must be overcome to make B7-H3-CAR-T therapy effective in treating brain tumors. One potential solution is the development of next-generation CARs and combination treatments with chemoradiation and biological agents 128.

NK cells are innate lymphocytes that lack antigen-specific receptors on T cells while expressing substantial neural cell adhesion molecule CD56, identifying and killing tumor cells, infected cells, and other abnormal cells. Studies have shown that B7-H3 has immunosuppressive effects on NK cells within the TME and in turn de-regulates NK cell-mediated anti-tumor effects 72. Recently, CAR-NK cells have been generated using the NK-92 cell line, a human non-Hodgkin's lymphoma NK cell line, for cancer immunotherapy to reverse the immunosuppressive TME and enhance the anti-tumor functions of NK cells. In GBMs and brain metastases, CAR-NK therapy targeting EGFRvIII or HER-2 has been explored in preclinical trials showing that CAR-NK therapy enhances activation of NK cells in the TME, induces anti-tumor effects by killing cancer cells and CSCs, and improves survival in rodent brain tumor models 129-131.

### Monoclonal and bispecific antibody

Monoclonal antibodies (mAbs) are an important component of anti-cancer therapy and are developed against tumor-expressed antigens. They function through different mechanisms such as direct binding to antigens, blocking antigen-antibody interactions, or mediating antibody-dependent cellular cytotoxicity (ADCC) 132. The successful application of mAb therapy targeting PD-1, PD-L1, and CTLA-4 for refractory cancers has encouraged the introduction of B7-H3 mAb therapy for tumors that express high levels of B7-H3.

Targeting B7-H3 therapy using mAbs has been shown to activate cytotoxic infiltrating immune cells, reorganize the intratumoral vascular network, enhance the sensitivity and clinical efficacy of chemotherapy, and inhibit tumor progression 85, 92, 133-135. Targeting B7-H3 with an mAb has demonstrated both safety and efficacy in the stage IV neuroblastoma 28. The murine IgG1 mAb, 8H9, which is specific for the principal form of B7-H3 (4IgB7-H3), shows promising effects for patients with B7-H3+ tumors and metastases to the central nervous system 136. In addition to the above-mentioned mouse mAb, a humanized mAb 8H9 targeting the FG loop of B7-H3 has been also designed and exhibits ADCC activity when co-cultured with peripheral blood mononuclear cells 137. MGA271, a fully humanized IgG1 B7-H3-targeting Fc-engineered mAb with increased activation affinity, enhanced anti-tumor function, and acceptable tolerance, and MJ18, an anti-mouse B7-H3-blocking mAb, have been reported to activate intratumoral anti-cancer immune function and inhibit tumor growth 138, 139.

In contrast to mAbs with only one binding domain, bispecific antibodies (bsAbs) are dual-affinity re-targeting proteins that are artificially generated with two binding specificities 140. BsAb thus recruits and induces cytotoxic immune cells to approach closely and kill tumor cells, regulate complementary anti-tumor signaling pathways, and mediates cancer cell immune escape. A recent study has shown that bispecific chemically self-assembling nanorings that target CD3 and B7-H3 increase T cell infiltration and facilitate selective cytotoxicity of B7-H3+ medulloblastoma spheroids independent of target MHC class I antigen 141. Furthermore, B7-H3/CD16 bispecific antibody (BsAb) has been shown to selectively activate NK cell activity, leading to enhanced antitumor efficacy mediated by NK cells 142. Additionally, B7-H3/4-1BB BsAb has been reported to boost the function of tumor-infiltrating differentiated CD8+ lymphocytes and enhance antitumor immune responses 143.

### Antibody-drug conjugates therapy

Antibody-drug conjugates (ADCs) are a novel therapeutic approach for refractory cancers that utilize a humanized mAb and cytotoxic drugs or radionucleotides. Preclinically developed ADC, MGC018, with a duocarmycin payload targeting B7-H3 has shown promising anti-tumor activity in preclinical models of various cancer types and has an acceptable safety profile in cynomolgus monkeys 38, 144. Another targeting ADC, DS-7300a, containing a B7-H3-targeting mAb and a DNA topoisomerase I inhibitor, exerts potent anti-tumor activities against B7-H3+ tumors and maintained acceptable pharmacokinetic and safety profiles in *in vitro* and *in vivo* models 145. Monomethyl auristatin E- and pyrrolobenzodiazepine-conjugated B7-H3-ADCs have been reported to impair tumor growth, induce tumor regression, and improve survival 40. In craniopharyngiomas, the B7-H3-targeted DM1-conjugated ADC exhibits tumor-suppression functions in both the traditional 2D cell culture model and the 3D organoid model 32. Therapy using B7-H3-mAb 8H9 labeled with ^131^I has shown efficacy and well tolerance in the treatment of recurrent metastatic neuroblastoma 146. Safety and anti-tumor effects of another ADC ^124^I-B7-H3 has been verified in a phase I trial for DIPGs 147. Three patients with embryonal tumor with multilayered rosettes have been treated with ^131^I-Omburtamab, which has been well-tolerated and shows potential therapeutic benefits following surgery and chemoradiation therapy 148.

## Current Understanding of B7-H3-related immunology and immunotherapy in Major Brain Tumor Types

### B7-H3 in gliomas

Gliomas are a heterogeneous group of tumors that arise from glia cells or neural stem cells, and are the most common and devastating primary malignant brain tumors in adults, accounting for around 50% of cases. A recent RNA-seq analysis of the TCGA and CGGA databases has revealed that B7-H3 has a higher mRNA level than any other B7 family members in gliomas 149. Immunostaining of B7-H3 showed that 86% of gliomas were B7-H3-positive, with the majority having moderate to high intensity 39. Results from a quantitative sandwich ELISA have revealed that sB7-H3 is present in the cerebrospinal fluid and blood serum of patients with gliomas 26. Clinically, the expression level of B7-H3 is correlated with the malignancy grade, molecular subtype, IDH mutation status, and patient prognosis 26, 27, 84, 126, 149. B7-H3 is selectively distributed in IDH-wildtype gliomas, mesenchymal gliomas, and high-grade gliomas, making it a potential immunotherapy-targeted biomarker in these refractory subtypes. Mechanistically, B7-H3 enhances proliferation and promotes invasion of glioma cells via activation of the JAK2/STAT3/Slug pathway, and induces EMT processes by downregulating E-cadherin and upregulating MMP-2/-9 protein 84.

Regarding newly-diagnosed GBMs, the most malignant and aggressive form of glioma, despite combined therapy including surgery, radiotherapy, and chemotherapy, the overall survival is only 14.6-16.0 months (20.9 months if with adjuvant tumor treating fields therapy), leading to one of the lowest 5-year survival of 6.8% among 33 human cancers 8. Almost all GBMs experience recurrence, and there is no established standard of care treatment for these relapsed lesions. Attention has increasingly shifted toward immunotherapeutic treatments targeting novel biomarkers. Among different glioma subtypes, the mRNA expression level of B7-H3 is the highest in GBMs, and the B7-H3 gene promoter is significantly hypomethylated as confirmed in the CGGA and TCGA datasets 149. B7-H3 is expressed both in the cytoplasm and at the cell membrane and is specifically upregulated in areas with hypoxia 23, 120. Specifically, 4IgB7-H3 is mainly detected in newly-diagnosed GBMs, while 2IgB7-H3 is specifically expressed in non-cancerous brain tissue and highly expressed in recurrent GBMs 23. Moreover, B7-H3 and the gene signature including GATA3 and immunosuppressive galactose-specific lectin 3 can help predict the prognosis of GBMs 150.

Several studies have attempted to apply CAR-T cell therapy targeting B7-H3 in GBMs *in vitro* and *in vivo* 24, 126, 127. The anti-tumor functions of B7-H3-CAR-T cells were confirmed in patient-derived GBM cells and GBM cell lines, and the survival rate of B7-H3-CAR-T-cell-treated GBM xenograft murine models and a clinical case was significantly improved 24, 25, 126.

### B7-H3 in meningiomas

Meningioma is the most common type of primary brain tumor in adults, with about 80% being benign and treated mainly through gross total surgical resection. However, a small percentage of meningiomas are classified as WHO grade 2 or 3 that are more likely to recur and require additional treatment such as adjuvant radiotherapy. No chemotherapy, targeted therapy, or immunotherapy has been approved for these types of malignancies. Recent studies have identified B7-H3 as the most highly expressed immune checkpoint in meningioma, with expression detected in 75%-100% of samples from both adults and children 30, 39. In a cohort of 234 B7-H3-positive meningiomas, high B7-H3 expression was associated with male sex, high-grade subtypes, and lack of adjuvant radiotherapy 30. B7-H3 was found to be expressed in almost all tumor cells in the majority of meningiomas, with elevated expression observed in tumors with gene mutations affecting the PI3K/AKT/mTOR pathway 31.

The protein phosphatase 2A (PP2A) inhibitor LB-100 has been shown to increase the expression of B7-H3 in malignant meningioma cells, making them more sensitive to B7-H3-targeted immunotherapy 151. Inhibition of nicotinamide phosphoribosyltransferase, which highly expresses in anaplastic meningiomas, suppressed tumor growth and inhibited B7-H3 expression by regulating STAT1, indicating its potential use as a combined treatment for B7-H3-targeted immunotherapy 152. The first-in-human study of B7-H3-targeted CAR-T cell therapy for treating a recurrent anaplastic meningioma showed a favorable safety profile, but revealed that B7-H3 expression decreased in the tumor part near the region of CAR-T-cell infiltration, requiring further investigation to address antigen loss and enhance CAR-T-cell trafficking 153.

### B7-H3 in DIPGs

DIPG is a type of malignant brainstem tumor that affects approximately 300 children in the United States every year. Unfortunately, due to its invasive nature and location, surgical removal is mostly not possible, and radiation therapy is the current standard of care. The median survival for DIPG is less than 12 months, making it a very deadly disease that requires innovative treatment approaches. Recent studies have found that B7-H3 immunoreactivity is present in 100% of DIPG specimens, and its mRNA expression level is significantly higher in DIPGs than in other non-diffuse brainstem gliomas 28.

The open-label, ongoing, first-in-human, BrainChild-03 phase I clinical trial is currently investigating the delivery of locoregional B7-H3-CAR-T cells to children and young adults with DIPGs (NCT04185038), and preliminary data from three DIPG patients revealed that repeated intracranial B7-H3 CAR-T-cell therapy induced local immune activation and persistent evidence of B7-H3-CAR-T cells in the cerebrospinal fluid 124.

### B7-H3 in ATRTs

ATRT is a rare but aggressive form of cancer that occurs in children under the age of three and originates in the brain or spinal cord due to genetic mutations in *SMARCB1* or *SMARCA4*. In the United States, approximately 60 children are diagnosed each year. Although multimodal therapies such as surgery, chemotherapy, and radiotherapy are available, the median survival time is only around 17 months. In a multicenter study, 100% of ATRT tumors and cell lines were found to express B7-H3, with 72% being strongly positive and 23% moderately positive 34. Another study also revealed that all ATRT specimens had B7-H3 immunostaining, with 92.3% exhibiting high and 7.6% showing medium staining intensities 39.

In mice models, CAR-T cells targeting B7-H3 administered via intracerebroventricular or intratumoral approaches showed faster kinetics, greater efficacy, and reduced systemic inflammation compared to systemic delivery of CAR-T cells administered intravenously. Additionally, locoregional CAR-T cell therapy induced antigen-specific protection from tumor relapse in both the brain and periphery regions 34.

### B7-H3 in medulloblastomas

Medulloblastoma is the most common malignant embryonal neuroepithelial tumor in children, accounting for 20% of pediatric brain tumors and 40% of posterior fossa tumors. It is classified into 4 molecular subgroups: WNT-activated, SHH-activated, Group-3, and Group-4, and clinical treatment typically involves neurological surgery, radiotherapy, and chemotherapy. The 5-year survival rate is 70%, affected by various factors such as tumor grade and type, patient age, overall health status, and treatment response. B7-H3 is expressed in almost all of pediatric medulloblastomas, and is particularly frequent in Group-4 tumors 35, 39, 154. In another study, the mRNA level of B7-H3 was found to differ significantly across molecular subgroups, and was significantly higher in tumor tissues than in non-tumor tissues. A higher B7-H3 level was associated with worse survival of patients with pediatric medulloblastomas 36.

Currently, B7-H3-CAR-T cell therapy for medulloblastoma is under investigation, as it has shown significant anti-tumor efficacy *in vitro* and regression of established medulloblastoma xenografts, particularly in cases where there is a high surface density of B7-H3 protein on the tumor tissues 122. Additionally, a bispecific chemically self-assembling nanoring that targets both CD3 and B7-H3 has been approved and shown to successfully cross the BBB and stimulate immune activation both intracranially and systemically in an orthotopic medulloblastoma model 141.

### B7-H3 in neuroblastoma

Neuroblastoma is the most common extracranial solid tumor of childhood, typically found in the peripheral nervous system and rarely inside the brain. Most tumors in children under one year of age are localized (stages 1-2), while older children often develop highly disseminated tumors with metastases involving the bone marrow, brain, liver, and skin. About half of neuroblastomas are classified as high-risk, with a 5-year survival rate of less than 50%. Despite treatments such as induction chemotherapy, surgery, autologous stem cell rescue, radiation therapy, isotretinoin, and targeted therapy against GD2, nearly 50% of patients with high-risk neuroblastomas relapse. B7-H3, similar to GD2, is highly expressed in both primary and metastatic neuroblastomas, and the immunoreactivity pattern is located on the cytoplasmic membrane and areas of rosette formation or ganglion differentiation 154, 155. High B7-H3 expression levels in neuroblastoma are associated with decreased survival rates, advanced tumor stages, and increased potential for metastasis 155-157.

In experiments, masking B7-H3 protein with the antibody (5B14) on isolated neuroblastoma cells enhanced NK cell-mediated lysis of tumor cells 37. Additionally, a combined treatment using artemether and doxorubicin was reported to suppress B7-H3 expression in neuroblastomas at both mRNA and protein levels, with artemether potentially serving as a therapeutic option via regulating B7-H3 for neuroblastomas 156. The B7-H3-targeting antibody-drug conjugate (ADC) m276-SL-PBD has shown promising results in neuroblastoma patients and cell line-derived mouse models, demonstrating an ultra-high treatment response rate and significant anti-tumor activity 158. Furthermore, bicistronic-CAR-T cells effectively eliminated neuroblastoma cells expressing GPC2 or B7-H3 and showed resistance to immune exhaustion compared to conventional CAR-T cells targeting a single antigen, indicating that this method may overcome heterogenous expression of targeted antigens in neuroblastomas 159. A recent phase I trial found that compartmental radioimmunotherapy with anti-B7-H3 monoclonal antibody administered intraventricularly had favorable dosimetry and significant survival benefits for neuroblastomas compared to historical data 160.

### B7-H3 in craniopharyngiomas

Craniopharyngiomas are benign neoplasms that develop in the sellar/parasellar region. There are two subtypes: adamantinomatous and papillary craniopharyngiomas. Despite being classified as a grade I benign brain tumor, craniopharyngiomas have a high recurrence rate, even after maximal surgical resection, due to their location near critical skull base structures and their aggressive growth pattern. The relative five-year survival rate is only 85.8%, the lowest among predominantly non-malignant brain tumors 161. Recent studies have found that B7-H3 is highly expressed in both subtypes of craniopharyngiomas, with more than 90% of tumors showing moderate-to-high-intensity staining, and is correlated with poor prognosis 33, 39, 162.

B7-H3/CD3 bi-specific T cell engagers have shown promising results *in vitro*, indicating that B7-H3-targeted therapy might be an effective treatment for refractory craniopharyngiomas 33. B7-H3-targeted CAR-T cell therapy has demonstrated potent tumor-killing effects in 2D cell culture models, but limited efficacy in 3D organoid models. However, the B7-H3 antibody-DM1 conjugate has exhibited tumor-suppressing function in both 2D and 3D models, providing a promising hint for ADC therapy for refractory craniopharyngiomas 32.

### B7-H3 in brain metastases

Brain metastases are common intracranial neoplasms that develop when cancer cells from other parts of the body, such as the lungs, breasts, or melanoma, spread to the brain. They affect about 20% of cancer patients and remain a significant cause of cancer-related deaths, despite the use of treatments such as surgery, radiotherapy, chemotherapy, and immunotherapy. Researchers are currently investigating promising treatment options that target novel markers. For instance, a study on metastatic melanoma *in vivo* and *in vitro* found that silencing B7-H3 reduced the ability of cancer cells to spread and increased symptom-free survival in mice and rats. The study also showed that B7-H3 regulated several genes associated with metastasis, including MMP-2, STAT3, IL-8, and TIMP-1 and -2 79. This finding is unique as it investigated the non-immunological function of B7-H3 in brain metastases. Another study has shown that co-expression of CCR2b in B7-H3-CAR-T cells significantly enhances the permeability of the BBB and increases the effectiveness of CAR-T cell therapy against NSCLC brain metastases both *in vivo* and *in vitro*. This study offers a new perspective on using engineered CAR-T cell therapy to control these lethal metastatic lesions 125.

## Ongoing Clinical Studies Targeting B7-H3 in Brain Tumors

Several early-phase clinical trials targeting B7-H3 in hematologic and other solid malignancies are ongoing, and some have revealed potent clinical efficacy and favorable tolerance (https://clinicaltrials.gov/). This suggests that novel effective immunotherapies, in addition to conventional treatments, may be available for refractory cancers 2, 5, 19, 98. In brain cancers, as demonstrated in preclinical studies and limited early-phase trial data, B7-H3 also acts as a promising therapeutic target and is being intensively evaluated in clinical trials. Ongoing clinical trials targeting B7-H3 in brain tumors are listed in **Table 1**.

### B7-H3-CAR-T cell therapy in brain tumors

In the field of CAR-T therapy, clinical trials targeting refractory CNS tumors, such as DIPGs, GBMs, and neuroblastomas, are ongoing to evaluate the safety, feasibility, pharmacokinetics, pharmacodynamics, and therapeutic efficacy of B7-H3-CAR-T therapy. There are seven ongoing trials (NCT04185038, NCT05474378, NCT05366179, NCT05241392, NCT04385173, NCT04077866, NCT04637503) being conducted in children or adult patients. In 2019, Seattle Children's Hospital initiated a non-randomized, parallel-assignment CAR-T-Cell locoregional immunotherapy for children and young adults with DIPGs and recurrent or refractory CNS tumors (NCT04185038). In this trial, eligible patients receive autologous T cells engineered to target B7-H3-expressing cells via an indwelling catheter placed in the tumor resection cavity or the ventricular system. The treatment options vary based on the location and type of tumors. Patients with supratentorial tumors will be assigned to arm A (tumor cavity infusion), patients with metastatic, leptomeningeal, or infratentorial tumors will be assigned to arm B (intraventricular infusion), and patients with DIPGs will be assigned to arm C (ventricular system infusion). Six clinical trials are also ongoing examining the safety and efficacy of B7-H3-CAR-T therapy for adult patients. Stanford University launched a trial last year that schedules B7-H3-CAR-T cells to be administered locoregionally in the ventricular system or in both the ventricular system and the resection cavity (NCT05474378). The primary outcome measures are the number of successful manufacturing products that meet the minimum assigned dose range, the maximum tolerated dose (MTD), and recommended phase II dose (RP2D). In another trial launched last year from the University of North Carolina Lineberger Comprehensive Cancer Center (NCT05366179), autologous B7-H3-CAR-T cells are only infused into the ventricular system of adult patients with relapsed or refractory GBMs. In this trial, a suspension of CAR-T cells (0.5 mL) is given via a Rickham catheter followed by a normal-saline flush (3-5 mL). The other four clinical trials are actively being conducted in China.

In a recently published study from Beijing Tiantan Hospital evaluating MTD and RP2D (NCT05241392), patients are treated with autologous B7-H3-CAR-T cells through an Ommaya device placed into the tumor resection cavity or ventricular system. Two trials are being conducted at the second affiliated hospital of Zhejiang University, with one assessing the pharmacokinetics and pharmacodynamics of CAR-T cell infusion in addition to its safety and clinical effectiveness (NCT04385173), and the other being a randomized, parallel-arm, phase I/II trial expected to enroll 40 patients randomized to receive B7-H3-CAR-T therapy during the adjuvant phase of Temozolomide chemotherapy or to undergo Temozolomide treatment alone (NCT04077866). The primary endpoint is overall survival, while adverse events, MTD, and multiple disease response parameters, including progression-free survival, are the secondary and other outcome measures. Finally, the trial from Shenzhen Geno-Immune Medical Institute utilizes multi-CAR-T cell therapy which targets disialoganglioside, prostate-specific membrane antigen, and B7-H3 in patients with relapsed and refractory neuroblastomas to assess the feasibility, safety, and efficacy of the “multiple targeting” approach and to assess its persistency in patients (NCT04637503).

### B7-H3-ADC therapy in brain tumors

ADC therapy is currently under investigation for the treatment of brain cancers. One clinical trial (NCT05063357) is examining the safety and efficacy of convection enhanced delivery of ^131^I-omburtamab, a murine IgG1 mAb that recognizes B7-H3 and is labeled with Iodine-131. Y-mAbs Therapeutics initiated this trial in 2021 to assess its effectiveness in treating children and young adults with DIPGs that have not progressed following external radiation therapy. Another phase II trial (NCT04743661), led by Memorial Sloan Kettering Cancer Center and Y-mAbs Therapeutics, is investigating the added utility of ^131^I-omburtamab in combination with irinotecan, temozolomide, and bevacizumab for patients with recurrent medulloblastoma. This trial will involve surgery, induction chemotherapy with irinotecan, temozolomide, and bevacizumab, and continued radioimmunotherapy with 2 doses of ^131^I-omburtamab. Patients with recurrent B7-H3+ ependymomas who have not responded to surgery, radiation, or other therapies will also be included in the study to assess the therapeutic efficacy of ^131^I-omburtamab. These two institutes have launched another clinical trial (NCT05064306) enrolling children and young adults who have CNS/leptomeningeal neoplasms to provide them with treatment with ^131^I-omburtamab. Additionally, a multicenter phase II/III trial (NCT03275402) is enrolling children with neuroblastomas and CNS/leptomeningeal metastases to receive up to 2 cycles of intracerebroventricular infusion with ^131^I-omburtamab to evaluate its clinical efficacy and safety. In addition to ^131^I-omburtamab, ^131^I-8H9 is also being investigated (NCT00089245) to assess its maximum tolerated dose in patients with refractory, recurrent, or advanced CNS/leptomeningeal cancers. ^177^Lu-DTPA-omburtamab has been tested in two trials (NCT04315246, NCT04167618) involving patients with leptomeningeal metastasis or medulloblastomas to evaluate its safety, pharmacokinetics, and therapeutic responses. However, these two clinical trials were recently withdrawn or terminated due to business priorities.

## Current Limitations and Challenges

Due to the low incidence of brain malignancies, enrolling a sufficient number of patients in clinical trials has been challenging. To address this issue, it is crucial for medical centers to collaborate and establish multi-center trials dedicated to the treatment of brain malignancies. By uniting efforts and resources, it will be possible to gather a larger patient population, facilitating the advancement of treatment options for these conditions.

While extensive research has been conducted on the potential ligands of B7-H3, no satisfactory results have been achieved thus far. To enhance the effectiveness of B7-H3 targeted therapy, it remains imperative to identify its binding partner and the downstream signaling pathway molecules associated with its function. Such discoveries would provide more specific and effective targets for therapeutic intervention.

Additionally, combination therapy has demonstrated superior efficacy compared to single treatment modalities in various brain malignancies. Incorporating different treatment approaches, such as focused ultrasound or tumor treating fields, alongside B7-H3 targeted therapy, holds promise as potential candidates for combination treatments. These modalities have the potential to enhance BBB penetration, overcome treatment resistance, and ultimately generate synergistic anti-tumor effects. Exploring these combination strategies could significantly improve the outcomes for patients with brain malignancies.

## Conclusions and Perspectives

B7-H3 is widely expressed on cancer cells and immune cells, playing a complex role in tumor progression and immune evasion. Although the crystal structure of B7-H3 has been confirmed, its specific binding receptors and downstream signaling pathways are still being investigated. B7-H3 functions as an immune co-inhibitor by suppressing the production of anti-tumor cytokines, inhibiting the activation and function of innate cytotoxic immune cells, and impairing T-cell immune response, thereby undermining adaptive immunity. Through interactions with infiltrating immune cells, tumor vasculature, and CAFs within the TME, B7-H3 establishes a pro-tumoral environment that supports uncontrolled tumor cell growth, accelerated metabolism, enhanced cancer stemness, and increased resistance to treatment. Recent advancements in understanding B7-H3's mechanisms of action and the rapid development of emerging treatment approaches have led to the investigation of various immunotherapies using CAR-T cells, CAR-NK cells, mAbs, bsAbs, and ADCs. Preclinical studies and early-phase clinical trials targeting B7-H3 in many refractory malignant brain tumor types have shown promising anti-tumor efficacy with limited toxicity. Currently, a growing number of trials focusing on B7-H3 targeted immunotherapy in brain malignancies, particularly GBMs, DIPGs, and brain metastases, are underway. These trials hold promise in providing new options and concepts for the treatment of these highly resistant types of brain tumors.

## Figures and Tables

**Table 1:** Active Clinical Trials Targeting B7-H3 in Patients with Brain Tumors.

Trial Number	Intervention	Phase	Launch Time	Settings	Sample Size	Patients	Pathology	Primary Endpoints
NCT04185038	CAR-T therapy	I	2019	Seattle Children's Hospital, US	90	Children and young adults	DIPGs and refractory or recurrent CNS tumors	Safety and feasibility
NCT05474378	CAR-T therapy	I	2022	Stanford University, US	39	Adults	Recurrent GBMs	Number of successful manufacturing products meeting the minimum dose range, the maximum tolerated dose, and the recommended phase II dose
NCT05366179	CAR-T therapy	I	2022	University of North Carolina, US	36	Adults	Relapsed or refractory GBMs	Safety and tolerability, cytokine release syndrome, and neurotoxicity
NCT05241392	CAR-T therapy	I	2022	Beijing Tiantan Hospital, China	30	Adults	Recurrent GBMs	Dose limiting toxicity and adverse events
NCT04385173	CAR-T therapy	I	2020	Zhejiang University, China	12	Adults	Recurrent or refractory GBMs	Adverse events, maximum tolerated dose, overall survival, and progression-free survival
NCT04077866	CAR-T therapy	I/II	2019	Zhejiang University, China	40	Adults	Recurrent or refractory GBMs	Overall survival
NCT04637503	CAR-T therapy	I/II	2020	Shenzhen Geno-Immune Medical Institute, China	100	Adults	Relapsed and refractory neuroblastoma	Adverse events
NCT05063357	ADC therapy	I	2021	Y-mAbs Therapeutics, US	36	Children and young adults	DIPGs that have not progressed following external beam radiation therapy.	Adverse events
NCT04743661	ADC therapy	II	2021	Memorial Sloan Kettering Cancer Center and Y-mAbs Therapeutics, US	62	Children and young adults	Recurrent medulloblastoma and ependymomas	2-year event free survival, and percentage of patients who met feasibility criteria in the ependymoma cohort
NCT05064306	ADC therapy	I	2021	Memorial Sloan Kettering Cancer Center and Y-mAbs Therapeutics, US	NA	Children and young adults	CNS/leptomeningeal neoplasms	NA
NCT03275402	ADC therapy	II/III	2017	Y-mAbs Therapeutics, US	52	Children	Neuroblastomas and CNS/leptomeningeal metastases	Overall survival rate at 3 years
NCT00089245	ADC therapy	I	2004	Y-mAbs Therapeutics, US	120	Children and adults	Refractory, recurrent, or advanced CNS/leptomeningeal cancers	Treatment related toxicities

## Abbreviations

AAMP: angio-associated migratory cell protein
ADC: antibody-drug conjugate
ADCC: antibody-dependent cellular cytotoxicity
ATRT: atypical teratoid/rhabdoid tumor
bsAbs: bispecific antibodies
CAF: cancer-associated fibroblast
CAR-T cell: chimeric antigen receptor T cell
CTLA-4: cytotoxic T lymphocyte-associated protein 4
DC: dendritic cell
DIPG: diffuse intrinsic pontine glioma
EMT: epithelial-to-mesenchymal transition
FOXP3: forkhead box P3
GBM: glioblastoma
IC: immune checkpoints
IFN-γ: interferon gamma
IL-4: interleukin-4
IL20RA: interleukin-20 receptor subunit α
JAK2: Janus kinase 2
mAbs: monoclonal antibodies
MMP-2: matrix metalloproteinase 2
MHC: major histocompatibility complex
NK cell: natural-killer cell
MTD: maximum tolerated dose
NSCLC: non-small cell lung cancer
PD-1: programmed cell death protein 1
PD-L1: programmed cell death ligand 1
PLA2R1: phospholipase A2 receptor 1
RP2D: recommended phase II dose
Th1 cell: T helper type 1 cell
Th2 cell: T helper type 2 cell
Th17 cell: T helper type 17 cell
TLR: toll-like receptor
TLT-2: TREM-like transcript 2
TME: tumor microenvironment
Treg cell: regulatory T cell
TREM: triggering receptor expressed on myeloid cell
scFv: single chain antibody fragment
STAT3: signal transducer and activator of transcription 3
VEGFA: vascular endothelial growth factor A
